# A Retrospective Study on the Use of Dermis Micrografts in Platelet-Rich Fibrin for the Resurfacing of Massive and Chronic Full-Thickness Burns

**DOI:** 10.1155/2019/8636079

**Published:** 2019-09-15

**Authors:** Alessandro Andreone, Daan den Hollander

**Affiliations:** ^1^Burns Unit, Inkosi Albert Luthuli Central Hospital, Durban, South Africa; ^2^University of KwaZulu-Natal, Durban, South Africa

## Abstract

The coverage of massive burns still represents a big challenge, even if several strategies are to date available to deal with this situation. In this study, we describe the use of a combination of platelet-rich fibrin and micrograft spray-on skin in order to increase the yield of grafted cells in patients. We treated a total of five patients, of which two were affected by massive burns and three with chronic burn wounds. Briefly, autologous micrografts were obtained by Rigenera technology using a class I medical device called Rigeneracons. The micrografts were then combined with PRF and sprayed on the wound bed by a Spraypen. Before applying PRF/micrograft spray-on skin, the wound bed was covered with an Integra® dermal template, and the wounds were dressed with a layer of antimicrobial dressing applied directly over the silicone layer. When the silicone layer of the dermal template started showing signs of separation, the wound was considered ready for grafting. In all cases, we observed a fast and complete skin graft on average after 7-10 days by PRF/micrograft spray-on skin treatment. In particular, two patients with massive burns reported rapid reepithelialization, and three patients with chronic burn wounds, two of whom had failed skin grafts before the procedure, had complete wound healing within a week. In conclusion, the results showed in this study suggest that the use of PRF/micrograft spray-on skin represents a promising approach in the management of burns or chronic burn wounds.

## 1. Introduction

The coverage of massive burns (i.e., more than 40% TBSA) is a challenge. As about 80% of the skin can be used as possible donor sites, the area to be grafted in massive burns often exceeds the available donor skin. A number of strategies are available to deal with this situation. Reharvesting from previously used donor sites is possible but requires a regeneration period of 2-3 weeks. Wide meshing (a ratio of 1 : 4 or more) and the MEEK technique have been proposed to make better use of available skin but also require time for reepithelialization of the interstices between the grafted areas, during which time these areas need to be protected from dehydration and microbial colonization. Cultured epithelial grafts also take about 3 weeks to be cultured, and although recently an affordable technique has been developed [1], long-term results remain poor. Common to all these strategies is that complete coverage of the burn wound takes time, during which period the patient is at risk of burn wound infection, sepsis, and death. In our environment, cadaver skin is rarely offered, and the high rates of HIV/AIDS preclude the use of amniotic membrane as a temporizing dressing. Spray-on skin cells (suspended epithelial cells) are an alternative for these patients [2, 3]. Although initially cells were cultured in a medium before being sprayed on the skin, recently a new technique has been described using fresh epidermal/dermal cells in suspension, which are sprayed on immediately after harvest [4, 5]. One problem with the use of spray-on cells has been that when the cells are suspended in a low-viscosity solution such as normal saline, they tend to be spread unevenly over the surface to be grafted, while there may be a significant graft loss, as cells float off the wound with the medium and end up on the towels. Some have combined spray-on cells with fibrin to deal with these problems [6, 7].

We recently developed a technique in which autologous micrografts are combined with platelet-rich fibrin and sprayed on the wound bed, in order to increase the yield of grafted cells. Although it is possible to use spray-on skin as the only skin cover, we have used the technique so far in combination with other grafting techniques, such as wide meshing using ratios of 3 : 1 or 6 : 1 or the MEEK technique, in accordance with the extension of the burn area requiring coverage and the available skin. The solution was first sprayed over the wound before application of the graft, followed by a second layer of solution. The spray was also applied to the donor site, which was further managed in the usual manner. A light compression bandage was used to protect the grafted areas. All grafted areas were reviewed on postoperative days 4-5 to assess graft take.

Autologous micrografts containing viable progenitor cells derived from the dermal-epidermal layer were obtained by Rigenera technology using the Rigeneracons device (Human Brain Wave, Italy), a biological tissue disruptor designed to cut tissues into small micrografts that are subsequently injected or sprayed into the desired area. This technology was originally used for the treatment of skeletal defects in orthodontic surgery [8, 9], but indications were subsequently extended to plastic surgery, in particular for the management of alopecia and chronic small wounds [10–12].

The combination of these two strategies, PRF and micrografts sprayed on the wound bed, has other potential advantages as well, related to the platelet-rich fibrin (abundance of growth factors, nutrition for developing epithelial cells), that made us explore its use in another group of patients which pose a problem in burns units, particularly under conditions of limited resources. Chronic burn wounds are the result of poor burn care, where deep burns are not excised, but the eschar is left to separate in the ward. This process can take anything between 3 and 8 weeks, at the end of which the wound is stuck in the inflammatory stage of wound healing, has very high levels of proteinases such as matrix metalloproteinases, and is often heavily colonized by microorganisms harbouring in biofilms [13]. Such wounds are characterized by epithelialization arrest and overgranulation, and sometimes a band of dark red inflamed tissue can be observed at the wound/skin interface. Such wounds have a high graft failure rate, and in the worst scenario, wound progression of the donor sites is observed, which may turn into full-thickness wounds. These wounds are a sign of poor burn care, which in a low-resource environment is often related to late referrals or to insufficient resources to timely take patients to theatre for a skin coverage procedure. The treatment of these wounds is difficult, necessitating a prolonged period of wound bed preparation before grafting can be attempted.

In this article, we describe the combined use of autologous micrografts in platelet-rich fibrin (PRF) to treat five consecutive patients affected by burns.

## 2. Patients and Methods

### 2.1. Ethics

Ethical approval for use of the Trauma/Burns database was granted by the UKZN-BREC ethics committee (Class Approval BE 207/09). Ethical approval for micrograft treatment was obtained from the Hospital Medical Human Research Ethics Committee, and all patients gave informed consent prior to the participation in this procedure.

### 2.2. Inclusion/Exclusion Criteria

The inclusion criteria were the following: age between 18 and 55 yrs., TBSA between 6 and 55% deep partial to full thickness with or without inhalational injury requiring split skin graft, and patients with open wounds not older than 2 months. The exclusion criteria were the following: patient younger than 17 and older than 55, wounds older than 2 months, TBSA less than 5% and more than 55%, and ventilated patient with septicaemia. A total of 12 patients were screened, and five patients were selected for the study.

### 2.3. Patients

We retrogradely analysed the records of patients treated with PRF/micrograft spray-on skin during the period between December 2017 and February 2018. Their clinical characteristics are reported in Table 1. All were men aged between 22 years and 46 years. Mechanism of injury was a flame burn in 4 and a hydrochloride acid burn in the fifth patient. The %TBSA burned ranged from 12% to 47%, with a mean of 22.5%.

The majority of the wounds in the patients with extensive burns were located over the torso, abdomen, neck, and face, and most of them had exposure of underlying bone structures postescharotomy. When a viable wound bed was achieved, large area wounds, including those involving the torso, the abdomen, and the neck and those involving an exposed bone (tibia), were then covered with an Integra® dermal template (Baroque, South Africa). The wounds were dressed with a layer of antimicrobial dressing (Acticoat® nanocrystalline silver dressing, Smith and Nephew) applied directly over the silicone layer followed by a nonocclusive dressing in four patients and negative wound pressure therapy in the remaining patient for 10 consecutive days. When the silicone layer of the dermal template started showing signs of separation, the wound was considered ready for grafting and taken to theatre. All the procedures performed on the patients before the PRF/micrograft spray application are indicated in Table 1.

### 2.4. Methods

**Figure 1:**
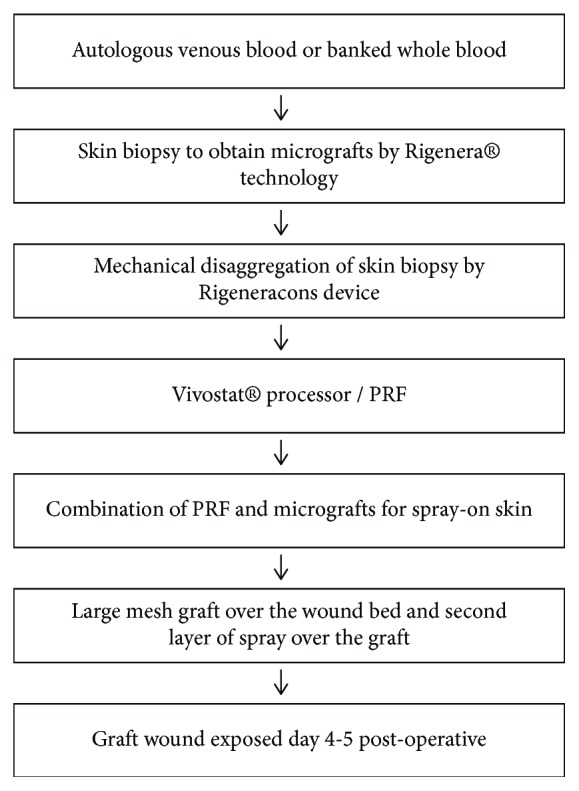
Sampling schedule and steps for the combined technique PRF/micrograft spray-on skin.

The method used in this study involves the combination of the following two techniques: platelet-rich fibrin (PRF) and micrograft spray-on skin. The steps are summarized in Figure 1 and detailed below.

### 2.5. Platelet-Rich Fibrin (PRF)

Vivostat® (Vivostat A/S, Lillerød, Denmark) technique [14] manufactures 5 ml of platelet-rich fibrin from 120 ml of the patient's own blood to which citrate is added to keep it in a liquid form, by ultracentrifugation for 25 minutes. The blood needs to be taken before the start of the surgery, as the resulting trauma will draw platelets to the operative site, diminishing the platelet concentration in the blood [15]. Activation of fibrin solidification does not require the addition of thrombin as in platelet-rich plasma but is simply accomplished by adding an alkaline buffer [16]. Where 120 ml of the patient's blood could not be provided, we have used banked whole blood to manufacture the PRF. The remainder of the blood can be transfused (back) into the patient. The centrifugate, consisting of platelet concentrate in polymerized fibrin, is then transferred to an application device (Spraypen), which sprays a fine mist of PRF over the wound. It was originally designed for use in neurosurgical procedures as a hemostatic agent to decrease postsurgical leakage and has successfully been used in renal [17] and thoracic surgery [18]. It was introduced into burn surgery to aid in hemostasis producing other benefits such as better graft fixation and higher graft take.

### 2.6. Micrograft Spray-On Skin

Micrografts were obtained using the Rigeneracons medical device as previously described [12]. Briefly, the donor area was prepared by gently removing the top epidermal layer using the Versajet® hydrodissector until the bleeding dermis tissue is reached. Subsequently, a thin layer of skin is harvested using a Davis® dermatome, obtaining a sample of 0.2 mm (0.008 inch) deep and 4 by 2 cm^2^ in size. The aim is to obtain cells from the epidermal-dermal junction [19]. This sample of the dermis is then cut with a scalpel blade into small pieces (1 to 2 mm wide) and inserted into the Rigeneracons device for mechanical disaggregation. This provides five milliliters of dermal micrograft suspension that is then transferred to a syringe provided in the Vivostat® kit. This syringe is then placed in the Vivostat® delivery unit alongside the syringe containing 5 ml of PRF solution. When added to the Vivostat® delivery system, both solutions are mixed immediately and are sprayed simultaneously over the wound bed using the Spraypen.

## 3. Results

**Figure 2:**
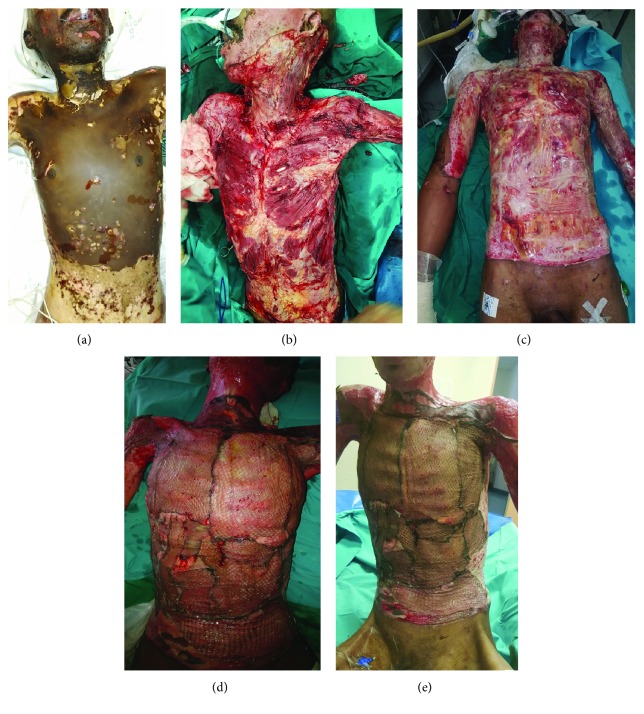
Representative images for patient 1. (a) Admission picture where a full-thickness burn over the anterior torso and neck is evidenced. (b) Post excision of burn eschar. (c) After application of Integra. (d) After application of PRF/spray-on skin; first dressing change: near-complete reepithelialization of grafted areas. (e) Results after 10 days postgrafting.

In the first patient, the grafted area was exposed on postoperative day 5 observing a perfect adherence of the graft onto the neodermis and visible new dermal formation in the meshed areas with a complete reepithelialization on day 7 post skin graft (Figures 2(c)–2(e)). We performed the same procedure over the remaining wounds 6 and 8 weeks later. The delay between the two surgeries resulted from complications as a result of tracheostomy for difficult intubation during a routine dressing change in theatre, which was further complicated by a pneumonia with bilateral pleural effusion. The patient was discharged after a total length of stay of 15 weeks.

In the second patient affected by chemical assault burn, we observed a good adhesion of the graft to the wound bed with new skin bridging formation visible in the meshed graft. The procedure was repeated 21 days later over the chest and the abdominal wall with similar results. Small additional wounds over the lower limbs and the feet were grafted a month after admission. The patient was discharged with a completely healed wound after a total hospital stay of 14 weeks.

**Figure 3:**
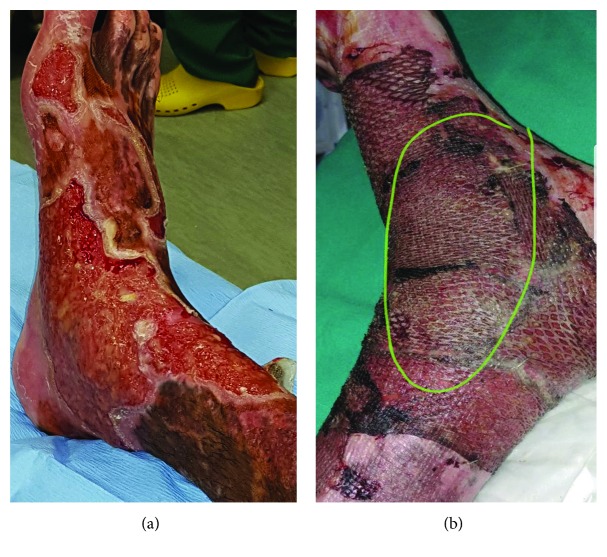
Representative images for patient 3. (a) Chronic burn wound with failed graft, overgranulation, and inflammatory margins. (b) Day 4 post widely meshed graft and PRF/spray-on skin where the wound is already reepithelialized.

In the third patient, at the first dressing change on day 4 postoperatively, the grafted areas were found to be perfectly healed and dry and were dressed only with aqueous cream (Figure 3). The donor areas (sprayed with the same combination) were fully healed when first exposed on day 6 post op. The patient was discharged on day 8 postoperatively after spending 13 days in the unit.

For the fourth patient, when exposed on day 5 post skin graft, 100% skin graft take was shown. The donor areas were found to be fully reepithelialized on day 6 post harvesting. The patient was discharged 20 days after admission with fully healed wounds.

In the fifth patient, grafted and donor areas were exposed on day 7 postoperatively. The donor areas were found completely healed, with excellent graft take over the grafted areas. The patient was discharged to base hospital 5 weeks after admission to our unit. A summary of clinical outcomes is reported in Table 2.

## 4. Discussion

Both the use of platelet-rich plasma (PRP) and of suspended epithelial cells are strategies that already form part of the armamentarium of the burn surgeon [20–22]. PRP is plasma with a higher concentration of platelets than baseline, obtained through centrifugation of the patient's blood, after which the supernatant is siphoned off [15]. The platelets are usually concentrated 3-5 times by this method, although the process has not been standardized [20]. Immediately before application, the solution is activated by the addition of bovine thrombin. When injected into the tissues or sprayed onto a wound surface, the activated PRP immediately forms a fibrin clot (similar to fibrin glue) resulting in hemostasis and fixation of the graft to the wound bed. Harkin et al. [5], Grant et al. [23], and Mittermayr et al. [6] demonstrated this in *pig* studies using a fibrin sealant, although others failed to demonstrate a beneficial effect on graft take [7]. The latter study was, however, criticized because only small wounds were used, and the dressings used did not represent what is normally used for massive burns [16]. Although initially used as an alternative to the fibrin sealant in corneal and nerve repairs, when PRP became commercially available in the 1990s, indications rapidly expanded to include its beneficial effects on tissue healing, regeneration, and cell proliferation. However, PRP has several disadvantages, not least of which is that it requires the addition of bovine thrombin with its resultant risks such as life-threatening coagulopathies and allergic reactions [22]. This led to the development of a second generation of platelet products, of which platelet-rich fibrin is the prime representative. PRF has proven to be as effective as PRP, while avoiding the side effects.

The advantage of a platelet-derived product such as PRF over fibrin sealants is, indeed, the presence of thrombocytes. It has long been recognized that platelets have many more functions than their role in coagulation [24, 25]. Platelets play a central role in pathogen surveillance and containment, as well as in wound healing. After activation, platelets release over 300 different proteins, including coagulation factor, chemokines, proinflammatory cytokines, and over 30 different growth factors, proteases, and protease inhibitors. In addition, platelets are able to modulate inflammatory and healing processes through direct cell-to-cell contact. They contain microbiocidal proteins and have been shown to engulf viruses and bacteria. The many growth factors produced by the platelet encourage multiplication as well as vascular ingrowth to provide nutrition to the seeded cells. Cell adhesion molecules released by platelets, such as fibronectin and vitronectin, enhance the migration of epithelial and vascular cells [26]. It has also been suggested that the fibrin strands may act as a template for epidermal migration [7]. Agren et al. [14] demonstrated that PRF, as obtained using the Vivostat technology, contains 3.9 times as many platelets as the baseline blood, as well increased levels of transforming growth factor-*β*1, platelet-derived growth factor-AB, basic fibroblast growth factor, and vascular endothelial growth factor. MMP-9 levels were reduced 139-fold. The beneficial effects of PRP on wound healing have been confirmed *in vitro* by Xian et al. [27] and in a murine study by Law et al. [28]. The effects of PRP seem to be concentration-dependent, with higher concentrations promoting inflammation and collagen deposition and lower concentrations enhancing wound remodelling [27]. A meta-analysis concluded that the application of PRP expedited the healing of wounds and in particular of chronic wounds [11]. However, a—theoretical—side effect may result from the intense inflammatory reaction caused by the increased platelet level, i.e., increased long-term scarring [29]. Venter et al. demonstrated that on its own, PRP has no benefit in full-thickness wounds. They explain this by the absence of epithelial cells (“substrate”) in the full-thickness burn [26].

In addition, the plasma contains nutrients for the epithelial cells during the period before the nutrient vessels have established themselves.

The use of epidermal cell suspensions (“spray-on skin”) in burn patients has been called a “common practice with no agreed protocol” with regard to indications for its use, techniques used, dressings to be used, timing of first wound review, and outcome measures [21]. In principle, two techniques are used. In cultured epidermal cell suspensions after enzymatic separation of the cells, they are brought into suspension and incubated for a minimum of 21 days to allow multiplication. The suspension is then sprayed over the wound bed or injected into the wound edges. The solution in which the keratinocytes are suspended varies from normal saline, lactate solution, hyaluronic acid matrix, to fibrin. Disadvantages of cultured epidermal cell suspensions are long delay caused by the culture, high rates of infection, and high costs and the fact that cultured cell suspensions contain only keratinocytes [30]. Long-term fragility of the new skin has also proven a problem [31]. Uncultured epidermal cell suspensions contain all types of skin cells, including keratinocytes, fibroblasts, melanocytes, and Langerhans cells and therefore should provide better long-term outcomes [4]. Wood et al. [19] analysed cell populations obtained with ReCell® and found that 75.5% of the total cell population was viable, which consisted of 64 ± 28.8% keratinocytes, 30.3 ± 14.0 fibroblasts, and 3.5 ± 0.5Y melanocytes. Separation of the skin cells may occur enzymatically (ReCell®) or mechanically (Rigenera®). No comparative studies are available to indicate which method is superior. A theoretical benefit of the Rigenera® method might be that keratinocytes and fibroblasts remain in contact, as cell-to-cell contact with fibroblasts has been suggested to be required for keratinocyte proliferation [27].

Zhao et al. [4] conducted a systematic review of the reported experience with spray-on skin in chronic wounds, including 5 studies and a total of 61 wounds. 44 (72%) of which had experienced (near) complete healing at the end of the observation period. Forty-three patients were managed with an uncultured suspension, with a good result in 30 (70%). The Medical Technologies Advisory Committee of the UK National Institute for Clinical Excellence (NICE) [3] published a review of the use of ReCell® in the acute management of burns. The review included a total of 817 patients in 3 published studies as well as an unknown number from congress abstracts (overlap between studies could not be excluded). Of the 817 published patients, 384 received epithelial cell suspension. Two studies found that the time to healing or total hospital stay was shorter for patients treated with epithelial cell suspension [32], while in the remaining study no statistically significant difference was found. Gravante et al. [2] also found no statistically significant differences for postoperative pain and development of contracture at one-month follow-up compared to patients who had a conventional skin graft.

We report here five cases in which we used a combination of PRF and micrograft suspension in the treatment of burn wounds. The first two patients had massive, deep burns, covering more than 40% of the body surface and in both cases “fourth degree” (extending into the subcutaneous tissues, in both cases penetrating through the anterior fascia of the intercostal muscles). Both patients were admitted to our Trauma Intensive Care Unit for ventilation of an associated inhalation injury. Both underwent early total excision of the burn wound, followed by application of a dermal substitute for two principal reasons. During the early stage, the dermal matrix in combination with the overlying polyurethane film provides a low-colonized, moist wound environment, allowing the wound to enter the wound healing stage of proliferation and the ingrowth of neovasculature into the collagen matrix of the dermal template. At the end of this stage, the film will lift off, a sign that the wound is now ready to receive a graft. At this point, the patient is taken back to theatre for a widely spaced skin graft (either a widely meshed graft or by the use of the Meek technique), supplemented by the PRF and spray-on micrografts. The use of spray-on epithelial cells over an Integra® dermal template was described in a porcine model by Wood et al. [33], and although these authors used a one-stage procedure, there have been no reports in humans.

In a resource-limited environment, a significant number of patients are referred late, sometimes after a period of many months in a peripheral hospital. This situation may result when the burns centres have insufficient beds to manage all burn patients in their catchment area, while regional hospitals lack resources and manpower to surgically manage burns. Burns that are referred late (i.e., more than a month post injury) have invariably full thickness, either because the depth of the wound was underestimated on initial assessment or the wound has progressed because of the heavy contamination with bacteria that inevitably occurs in chronic burn wounds. Such burns are characterized by biofilms and high concentrations of natural proteinases, such as matrix metalloproteinases (MMPs), which lock the healing process in the inflammatory stage. Split skin grafts will often fail on such wounds, and this may be associated with progression of the donor sites to full-thickness wounds, eventually leaving a larger area to cover than before the attempted skin graft. Two of the three patients in our series had failed skin grafts before the index procedure. We currently treat these wounds with Prontosan® in combination with either nanocrystalline silver or honey-based dressings in an attempt to break down biofilms and decrease the bioburden and with topical steroids to reduce MMPs and other inflammatory mediators. This usually results in a graftable wound bed in 1-2 weeks, when the patient is taken to theatre. The three patients described underwent this regimen of wound bed preparation and were taken to theatre when the surgeon deemed the wounds ready for grafting. They were then covered with widely meshed graft and PRF/spray-on micrografts, each one with excellent results.

Although we originally thought we were the first to use this combination in humans, we have since come across a report from Birmingham, UK, where a similar protocol, but using the ReCell technique of epithelial cell preparation, was used to cover a 15% area of full-thickness burn over the back [16]. The patient had previously undergone five grafts, associated with significant graft loss. In the sixth sitting, a mixture of platelet-rich fibrin and suspended epithelial cells (processed using the ReCell® technique) was applied using the Vivostat® system. On day 7, there was excellent graft take.

Being a small retrospective evaluation, this study has several limitations. In the two patients with massive burns, although the graft take was excellent and sustained, the length of hospital stay in both patients was still longer than expected from the one day/%TBSA burnt rule, which on average applied to our patients in a previous epidemiological study [34]. The short follow-up period did not allow for assessment of long-term results, which might be adversely affected by the platelets administered to the wound as stated above. The patients in which the technique was used for the management of chronic wounds with reepithelialization arrest were subjected to a multimodal treatment, of which very few of the components have been subjected to scientific investigation. However, we feel that the results of PRF/micrograft spray-on skin are sufficiently promising to warrant setting up a randomized trial and setting one up.

## 5. Conclusion

The use of a micrograft suspension in platelet-rich fibrin was described. Two patients with massive burns who were subjected to wide meshing experienced rapid reepithelialization, which however was not translated to a shorter stay in the hospital. Three patients with chronic burn wounds, two of whom had failed skin grafts before the procedure, had complete wound healing within a week. Further investigation in the form of a properly executed randomized trial is warranted.

## Figures and Tables

**Table 1:** Clinical characteristics of five patients enrolled with the indications of previous treatments and application of PRF/micrograft spray-on skin.

Patient	Age	Case history	Previous treatment	Treatment with PRF/micrograft spray
1	39	Assault with an unknown flammable substance, resulting in 45% TBSA full-thickness burn over the anterior torso and neck and inhalational injury (Figure 2(a))	Full necrotomy by performing sharp excision and hydrosurgery with following exposure of the ribs and sternum (Figure 2(b)). Coverage of wound bed with a dermal template (Integra®) and an antimicrobial dressing (Acticoat®, S&N) (Figure 2(c))	After 10 days, the patient was taken to theatre again where the silicone layer over the chest and the flank was removed and a 4 : 1 meshed graft was placed over the neodermis. PRP/micrografts were sprayed over the wound bed and over the meshed graft. The grafted area was exposed on postoperative day 5.

2	22	Full-thickness chemical assault burn with 25% TBSA. The clinical picture suggested a hydrochloride acid burn and the areas involved were the chest, abdomen, multiple areas over the face, both arms, and both legs.	Complete excision of the burn area was performed down to the periosteum of the ribs. The wound bed was covered with an Integra® dermal template and an antimicrobial dressing (Acticoat®, S&N).	After 8 days, the silicone layer was lifted and removed, both arms and forearms were grafted with a mesh graft to a ratio of 4 : 1; PRF/micrografts were sprayed over the bed and the graft. The grafted areas were exposed on postoperative day 5.

3	43	Full-thickness poured petrol burn over both lower limb and feet with a 6.5% TBSA	Conservative treatment with silver sulfadiazine dressings on alternative days for over 3 months. Due to nonhealing wounds over the affected areas, a pus swab was done showing a wound colonization with *Pseudomonas aeruginosa*, responsive to ciprofloxacin.	After a 5-day course of antibiotics, he was taken to theatre. His wounds were debrided with hydrosurgery (Versajet®), and a combination of PRF and micrografts was sprayed over the wound bed and over the meshed graft (3 : 1).

4	45	Full-thickness flame burn with 15% TBSA treated by a traditional healer. The burn involved the left flank, part of the chest, left arm, and forearm.	Conservative treatment for 3 months before transfer to the Burn Unit where the patient was dressed with polyurethane foam (Biatain®, Coloplast)	After 4 days, wounds were debrided with the Versajet® hydrodissector and wound bed was sprayed with PRF and micrografts. Harvested skin was meshed 4 : 1 and applied, followed by a second application of PRF/micrografts. A dressing with plain petrolatum gauze was performed.

5	33	Electrical burns with 15% TBSA on both arms and both legs. He had been found unconscious after the arc of electricity hit the ladder he was holding (±66000 V).	Conservative treatment for 2 months before transfer to the Burn Unit	The wounds were cleansed with hydrosurgery (Versajet®) and then PRF/micrografts were sprayed. Ultrathin layer Integra® was then applied over the patella and the popliteal fossa on the right leg, and the areas were then covered using a modified Meek technique (Humeca®, Netherlands).

**Table 2:** Clinical outcomes.

Patient	Age	Case history	Graft take	Full wound closure
1	39	Assault with an unknown flammable substance, resulting in 45% TBSA full-thickness burn over the anterior torso and neck and inhalational injury (Figure 2(a))	97%	D/C 15 weeks post grafting
2	22	Full-thickness chemical assault burn with 25% TBSA. The clinical picture suggested a hydrochloride acid burn, and the areas involved were the chest, abdomen, multiple areas over the face, both arms, and both legs.	96%	D/C 9 weeks post grafting
3	43	Full-thickness poured petrol burn over both lower limb and feet with a 6.5% TBSA	98%	D/C 22 days post grafting
4	45	Full-thickness flame burn with 15% TBSA treated by a traditional healer. The burn involved the left flank, part of the chest, left arm, and forearm.	98%	D/C 39 days post grafting
5	33	Electrical burns with 15% TBSA on both arms and both legs. He had been found unconscious after the arc of electricity hit the ladder he was holding (±66000 V).	98%	D/C 32 days post grafting

## Data Availability

The data used to support the findings of this study are included within the article.
